# Contribution of *Streptococcus anginosus* to Infections Caused by Groups C and G Streptococci, Southern India

**DOI:** 10.3201/eid1604.090448

**Published:** 2010-04

**Authors:** Silvana Reißmann, Claudia Friedrichs, Reena Rajkumari, Andreas Itzek, Marcus Fulde, Arne C. Rodloff, Kootallur N. Brahmadathan, Gursharan S. Chhatwal, D. Patric Nitsche-Schmitz

**Affiliations:** Helmholtz Centre for Infection Research, Braunschweig, Germany (S. Reißmann, A. Itzek, M. Fulde, G.S. Chhatwal, D.P. Nitsche-Schmitz); University Hospital Leipzig, Leipzig, Germany (C. Friedrichs, A.C. Rodloff); Christian Medical College, Vellore, Tamil Nadu, India (R. Rajkumari, K.N. Brahmadathan)

**Keywords:** Streptococcus anginosus, streptococci, groups C and G streptococci, suppurative infections, pharyngitis, bacteria, abscesses, research

## Abstract

Vellore, a region in southern India, has a high incidence of severe human infections with β-hemolytic group C and G streptococci (GCGS). To determine the causative species in these infections, we conducted 16S rRNA gene sequencing: *Streptococcus dysgalactiae* subsp*. equisimilis* (81%) and *S. anginosus* (19%) were the causative organisms in the 2-year study period (2006–2007). We used PCR to detect the virulence-related *emm* gene; results showed that it was restricted to *S. dysgalactieae* subsp. *equisimilis* isolates of 99.2% tested positive. Due to a novel marker, *S. anginosus* and *S.* c*onstellatus* can be quickly and accurately distinguished from other members of the genus. The notable contribution of the anginosus group to human infections suggests that this group of obligate pathogens deserves more attention in healthcare and research.

## Abstract 2

This neglected pathogen causes a large portion of these infections.

Group C and group G streptococci (together GCGS) were first recognized as human pathogens in 1935 by Lancefield and Hare (*1*). Since then, awareness about their importance has greatly increased, especially within recent years (*2**–**6*). Similar to infections with *Streptococcus pyogenes,* the prime example of a pyogenic streptococcal pathogen, infections with GCGS can develop into life-threatening necrotizing fasciitis, sepsis, and streptococcal toxic shock–like syndrome. Lancefield groups C and G comprise a variety of species; one of those species, *Streptococcus dysgalactiae* subsp. *equisimilis* (SDSE), frequently causes human infections. This species can cause the whole spectrum of infections caused by *S. pyogenes* (*4**,**5*).

SDSE likely owes its virulence in humans to homologs of prominent *S. pyogenes* virulence genes (*7**,**8*). Most SDSE strains isolated from human infections possess *emm* genes (*9**,**10*), which code for the potent virulence factor called M protein (*11*). This surface localized protein contributes substantially toward the virulence of both *S. pyogenes* and SDSE in human hosts because it acts as an adhesin, invasin, and antiphagocytic factor (*11*). Strain-to-strain variability in the N terminus of M proteins, driven by the adaptive immune response of the host, has led to a vast *emm* type diversity. More than 100 genetically distinct M proteins exist within the GCGS group and form the basis for *emm* genotyping (*12*). However, SDSE is not the only species that causes severe diseases in humans. The variety of GCGS includes the typical animal pathogens *S. equi* subsp. *zooepidemicus* (group C) and *S. canis* (group G), which have the potential to cause zoonotic infections (*13**,**14*). Other streptococcal species that are pathogenic in humans and that occasionally expose groups C and G carbohydrates are gathered under the umbrella term anginosus group (*15*).

In the literature, the designation *S. milleri* (*16*) has often been used for streptococci of this group, although it has never been an officially approved name (*15*). Streptococci of the anginosus group can reside commensally in the human oral cavity but have a certain propensity to cause pharyngitis, bacteremia, and serious purulent infections in the deep neck and soft tissue and in internal organs such as the brain, lung, and liver (*17**–**25*). The bacteria cause severe infections after surgical treatments and infect implanted material, thereby posing a problem of substantial clinical relevance (*20**,**26**,**27*). The species diversity within the GCGS highlights the limits of Lancefield grouping by agglutination assays, the typically applied method in the diagnosis of streptococcal infections. The genetically distinct GCGS species differ in pathogenesis, virulence mechanisms, and antimicrobial drug susceptibility. Thus, finding the optimal treatment regimen can be facilitated by species determination. Diagnosis of anginosus group infections is particularly difficult. The group comprises the species *S. anginosus*, *S. intermedius,* and *S. constellatus* of which the 2 subspecies, *S. constellatus* subsp. *constellatus* and *S. constellatus* subsp. *pharyngis*, are further distinguished. Identification of the anginosus group is complicated by wide phenotypic and antigenic diversity, even within 1 species. Although most anginosus group isolates belong to the non-β-hemolytic oral streptococci, β-hemolytic strains are found in all 3 species. Some anginosus group strains carry a typeable Lancefield group antigen, which belongs to group F, C, G, or A (*28*).

Routine microbiologic diagnosis of streptococcal infections is often restricted to determination of the type of hemolysis and of the Lancefield group. Identification of streptococci to the species level is rarely carried out. This leaves a considerable risk for misidentification of causative pathogens, which can lead to an inappropriate treatment of the infection (*29**–**31*). As a further consequence of the complications associated with species determination, insight into the epidemiology of infections with certain streptococci remains imprecise, and the epidemiology of the anginosus group, in particular, remains widely elusive. Comprehensive insights are missing that could enable clinicians to reevaluate respective diagnostic and therapeutic routines. Moreover, such insights may stimulate research that aims at clarifying the pathogenesis of these streptococcal species and the development of specialized treatments and prevention strategies. These goals have motivated our cross-species study of human pathogenic GCGS from Vellore, India, a region with a high incidence of such infections. Examination of epidemiologic contributions of the different streptococcal species was combined with a cross-species screening for *emm* genes, which identified a novel gene in *S. anginosus* and *S. constellatus*. The use of this gene as a marker for fast detection of infections caused by these 2 streptococcal species was investigated.

## Methods

### Bacterial Strains, Lancefield Typing, and Genomic DNA

Clinical isolates of group G and group C streptococci were collected at the Department of Clinical Microbiology, Christian Medical College, Vellore, India, from 2006 through 2007. The collection comprised isolates from patients with pharyngitis (throat swabs), patients with respiratory (sputum) and urinary tract infections (urine), and from other suppurative foci (pus) and blood. Streptococci were collected from 2004 through 2006 at the University Hospital in Leipzig, Germany. The isolates were recovered from blood cultures, wound swabs, aspirates of peritonsillar abscesses, abscesses in the inner body, and catheter tips. The collection was typed as described previously (*32*). Bacterial strains were subcultured on Columbia agar with 5% sheep blood (Becton-Dickinson, Franklin Lakes, NJ, USA). Cultures from single colonies were grown overnight (37°C in 5% CO_2_) in Todd-Hewitt broth (Becton-Dickinson) supplemented with 0.5% yeast extract. The Lancefield group was determined by using the Slidex streptococcal grouping kit (Oxoid, Basingstoke, UK). Genomic DNA was isolated by using the DNeasy Blood and Tissue Kit (QIAGEN, Hilden, Germany) according to the manufacturer’s protocol with a minor variation: the incubation with proteinase K was carried out at 70°C for 30 min.

### Sequencing of *emm* and 16S rRNA Genes

Amplification of *emm* genes from streptococcal genomic DNA samples was performed by using the primers 1 and 2 recommended by the Centers for Disease Control and Prevention (*12*) (*emm*-PCR). To amplify the 16S rRNA gene, PCR was performed with a pair of generic primers: 16S rDNA fwd and 16S rDNA rev (Table 1), for gram-positive bacteria as described (*33*). PCR experiments were analyzed by agarose (1%) gel electrophoresis. PCR products were purified by using the QIAGEN PCR purification Kit (QIAGEN, Hilden, Germany) and sequenced by using primer 16S rDNA fwd (Table 1), the Big Dye Terminator reaction, and an ABI Prism 377 system (Applied Biosystems, Foster City, CA, USA).

**Table 1:** Sequences of primers used in study of groups C and G streptococci, Vellore, India, and Leipzig, Germany*

Name	Sequence (5′ → 3′)	Application
16S rDNA fwd	AGAGTTTGATCCTGGCTC	16S rDNA amplification
16S rDNA rev	GGTTACCTTGTTACGACTT	
Primer 1	TATTCGCTTAGAAAATTAA	*emm* amplification
Primer 2	GCAAGTTCTTCAGCTTGTTT	
M13 rev	CAATTTCACACAGGAAACAGCTATGAC	Sequencing of 1.1-kb fragment of *moac*
M13 fwd	GTAAAACGACGGCCAGTGAATTG	
*moac*1	CAAGGAATTGATTCAGCAACAGTGC	Inverse PCR and sequencing of *moac*
*moac*3	CTTCTCAACAAGCATTGGCAGATGC	
*moac*6	GTGTGTATACACGTCGGACATTTCC	
*moac*7	GGTACAGTAATGGGAAGTTTGTTAGG	
*moac*8	GCGGATTGACTTCATTTGGCGTCG	
*moac*9	GGTTTGGGGATGTCTTCTTCCATGG	
*moac*10	GCATCTCAAATCAGACGAGCAAGC	
*moac*11	CTTGAACTTGTCTTCGCATGGAGC	
*moac*12	GACTATTATCAAACGGTATTTGCTCG	
*moac*2	CCAATTCACTTGAATTGACGAATCC	
*moac*4	GCCCAACCTGAAGACAGTTGAGC	
*moac*5	CTGACGAAAAGAGAGCCAGATATCC	
*moac*13	CTGATACCATAATCTGACATCACTGC	
*moac*14	GAAGTTGAACTATCTCCAATCACCG	
*moac*-SP	ATGAAAAAATCCATTCTAAATAAGGATATC	Screening for 3,272-bp fragment of *moac*
*moac*-TMH7	AAGACTGGCACAAGATATAC	
*moac*-BamH1	GCGGATCCGGTCATTTTCCAAGCAAGG	Screening for 962-bp fragment of *moac*
*moac*-Sal1	GCTGTCGACTTATTAAATTCAGCCTGCTTTTTCTCC	

*moac, marker of *Streptococcus anginosus* and *S. constellatus.*

### Inverse PCR and Sequencing of a Marker of *S. anginosus* and *S. constellatus*

Application of *emm*-PCR on the genomic DNA of *S. anginosus* strain SV52 produced a 1.1-kb amplicon, subsequently identified as a fragment of a marker of *S. anginosus* and *S. constellatus* (*moac*). The fragment was cloned into the pCR2.1-TOPO vector by using the TOPO TA cloning kit (Invitrogen, Carlsbad, CA, USA) and then sequenced by using primers M13 rev and M13 fwd (Table 1). Inverse PCR was used to amplify the genomic DNA segments flanking the 1.1-kb fragment of *moac*. Genomic DNA (1 µg) was digested separately with 1–3 of the following enzymes: *Ase*I, *Avr*II, *Bam*HI, *Bgl*II, *Bsa*I, *Bse*YI, *Eco*RI, *Hind*III, *Nde*I, *Nsi*I, *Pst*I, *Sac*I, *Sal*I, *Spe*I, *Xba*I, *Xho*I (New England Biolabs, Ipswich, MA, USA). Digestion was carried out for 16 h under conditions recommended by the manufacturer. Digested genomic DNA was diluted both 100-fold and 10-fold before self-ligation with 160 U ligase (16 h, 16°C). Afterwards the ligations were used as a template in PCR experiments with different combinations of the primers moac1 to moac14 (Table 1). PCR was performed in a thermocycler (Biometra GmbH, Goettingen, Germany) with an initial denaturation step (4 min at 96°C), followed by 30 cycles of denaturation (40 s at 94°C), annealing (30 s at 56°C), and extension (1 min, 30 s, up to 3 min, 72°C). A final extension was carried out for 5 min at 72°C. PCR products were analyzed, purified, and sequenced as described above.

### Screening for *moac*

Isolated genomic DNA was tested by PCR for the presence of the *moac* gene. For this purpose, 2 primer pairs (Table 1) were used to amplify a 3,272-bp fragment (primers: *moac*-SP, *moac*-TMH7) and a 962-bp internal fragment (primers: *moac*-*Bam*HI, *moac*-*Sal*I) of *moac*, respectively. For amplification of the 962-bp fragment, initial denaturation (4 min at 96°C) was followed by 25 cycles of denaturation (40 s at 94°C), annealing (30 s at 53°C), and extension (1 min 30 s at 72°C), and then by a final extension step for 5 min at 72°C. The 3,272-bp fragment was amplified with 30 cycles by using an annealing temperature of 50°C and an extension time of 3 min, 20 s. The PCR products were analyzed, purified, and sequenced as described above.

### Sequence Analysis

Sequence data were processed and analyzed by using the software BioEdit version 7.0.1 (Isis Pharmaceuticals, Carlsbad, CA, USA). Coiled coil structures and transmembrane helixes were predicted with the Web-based programs Coils (*34*) and TMHMM Server v. 2.0 (*35*), respectively.

## Results

### Typing of GCGS Isolates and Screening for *emm* (-like) Genes

The Vellore region in India has a high incidence of group C and G streptococcal infections (*36*). For characterization of local pathogenic GCGS strains, 313 isolates were collected from patients with suppurative or invasive infections at the Christian Medical College. The study was designed to be cross-species; therefore, the only preselection criteria applied were type of hemolysis and Lancefield type. Sequencing showed that 254 of the strains showed highest homology with the 16S rRNA gene from SDSE and that the sequences of the remaining 59 strains were homologous to *S. anginosus* (Table 2). Notably, all *S. anginosus* strains belonged to Lancefield group G. M proteins have a fundamental role in streptococcal infections. Therefore, and because of a potential power to discriminate between SDSE and anginosus group strains, the collection was examined by *emm*-PCR (*12*) (Table 2). Specific PCR products were obtained for 252 of the 313 stains. Two of the SDSE strains and the 59 anginosus group strains were not *emm*-typeable. The SDSE strains comprised 44 different *emm* types, of which 2, stG120 and stG351, have not been previously described (Table 3).

**Table 2:** Typing of groups C and G streptococci collection, Vellore, India

Species (N = 313)	16S rRNA gene	*emm*-PCR positive/negative	*moac*-PCR* positive/negative	Lancefield group	
				G	C
*S. dysgalactiae* subsp. *equisimilis*	254	252/2	0/254	208	46
*S. anginosus*	59	0/59	59/0	59	0

*Tested with 2 primer pairs (Figure). *moac,* marker of *Streptococcus anginosus* and *S. constellatus.*

**Table 3:** *emm t*yping of pathogenic *Streptococcus dysgalactiae* subsp. *equisimilis* isolates from Vellore, India*

*emm t*ype	No. strains
stG245	32
stG6792	18
stG643	17
stG6	14
stG653	14
stC1400	12
stGL265	12
stG652	11
stG866	10
stC5345	9
stG485	9
stC74a	9
stC6979	8
stC839	7
stGLP1	7
stGrobn	5
stC922	4
stG211	4
stC2sk	3
stG2574	3
stG4222	3
stG480	3
stG4831	3
stG5420	3
stG7882	3
stG120*	3
*emm*31	2
stC36	2
stCK401	2
stG166b	2
stG2078	2
stG840	2
stG97	2
stG10	2
stG351*	1
*emm*23	1
stC1741	1
stC3852	1
stC46	1
stC5344	1
stC6746	1
stC-NSRT2	1
stGM220	1
stMD216	1

*The 5′ sequences of newly identified *emm*-genes are deposited in GenBank (accession nos. FJ036933, FJ036936, and FJ036937) and in the *emm*-gene database of the Centers for Disease Control and Prevention, Atlanta, GA, USA.

### Characteristics of a Newly Discovered Open Reading Frame of *S. anginosus*

Our recent survey at the University Hospital in Leipzig, comprising 127 cases of severe infections with oral streptococci, found that a large number of infections were caused by strains that belonged to the anginosus group (26%). We included the anginosus group strains from the Leipzig collection in the *emm-*typing experiments described above to increase the number of isolates and the phenotypic diversity of the collection in terms of Lancefield antigen and type of hemolysis. Like the *S. anginosus* strains from Vellore, none of the 33 strains from Leipzig was *emm*-typeable. Under less stringent conditions, the PCR produced a low concentration amplicon of 1.1 kb (Figure).

**Figure:**
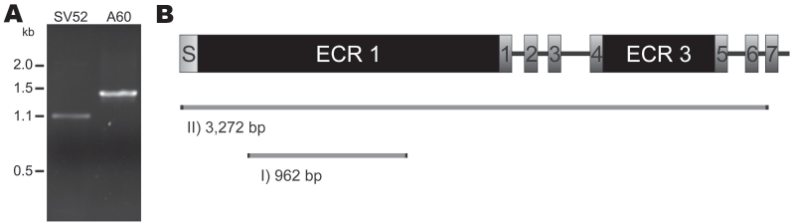
Amplification and characterization of a newly discovered open reading frame (ORF) of *Streptococcus anginosus*. *A*) Gel electrophoresis after *emm*-PCR on *S. anginosus* isolate SV52 (*SV52*) and *S. pyogenes* strain A60 (*A60*). The latter isolate was used as a control that possesses an *emm*3 gene. The *S. anginosus* strain generated a low concentration 1.1-kb amplicon, as compared with the 1.4-kb product of the *S. pyogenes* strain. Inverse PCR based on the 1.1-kb sequence of SV52 showed an ORF of 3,363 bp. Its sequence is predicted to code for the membrane protein that is schematically depicted in panel B*.* It comprises an N terminal signal peptide (S) followed by a large extracellular region of 60 kDa (ECR1), 7 transmembrane helixes (1–7), and another large extracellular region of 23 kDa situated between the fourth and the fifth transmembrane helix (ECR3). Positions of the 2 alternative marker of *Streptococcus anginosus* and *S. constellatus* PCR products (I and II) relative to the full-length sequence and their length are indicated in basepairs.

Sequencing of the of 1.1-kb PCR product did not show considerable similarities with *emm*-genes, however, the lack of stop codons in 1 frame of translation motivated further investigations on that PCR product. Inverted PCR experiments on *S. anginosus* strain SV52 identified an open reading frame (ORF) of 3,363-bp (GenBank accession no. GQ456155). Computational analysis predicts a 124-kDa membrane protein with 7 transmembrane sequences and a signal peptide for secretion (Figure). The predicted protein further consists of 2 larger extracellular regions, one of 23 kDa located between the fourth and fifth transmembrane sequence and one of 60 kDa at its N terminal end. The central part of the N terminal extracellular region contains a stretch of heptad-repeats (aa 204 to 520 of the mature protein), which may enable coiled-coil oligomerization. Other than this, the protein has no obvious or significant features of an M- or M-related protein. Prediction of 7 transmembrane sequences suggests a receptor function or a function in transport processes. The latter assumption is corroborated by a high similarity of the protein sequence to numerous bacterial permease components of ATP-binding cassette transporters.

### Distinguishing of *S. anginosus* and *S. constellatus* from Other Oral Streptococci by a Newly Discovered ORF

The distribution of the newly discovered ORF in the Leipzig collection of oral streptococci was examined by PCR with 2 different primer pairs (Table 1). Both primer combinations gave identical results. The collection consists of 127 clinical isolates of which 33 belong to the anginosus group (17 *S. anginosus*, 8 *S. constellatus* subsp. *constellatus*, 4 *S. constellatus* subsp. *pharyngis*, 4 *S. intermedius*); 78 belong to species of the mitis group (*S. mitis*, *S. oralis*, *S. sanguinis*, *S. parasanguinis*). Fourteen strains have been typed as *S. salivarius* and 2 as *S. gallolyticus*. Specific PCR products were obtained exclusively within the anginosus group. All *S. anginosus* and *S. constellatus* isolates tested positive. Negative PCR distinguished *S. intermedius* from the other 2 species. The results were confirmed in experiments with reference strains from the Deutsche Sammlung für Mikroorganismen und Zellkulturen (Table 4). Taken together, the results demonstrate that the newly discovered gene is a marker that discriminates *S. anginosus* and *S. constellatus* from other oral streptococci. The gene was therefore designated *moac*.

**Table 4:** Distribution of *moac* within a collection of oral streptococci, Vellore, India, and Leipzig, Germany*

Group/species	No. strains	*moac*-PCR
Anginosus group	33	
*Streptococcus anginosus*	17	+
*S. constellatus* subsp. *constellatus*	8	+
*S. constellatus* subsp. *pharyngis*	4	+
*S. intermedius*	4	–
Bovis group	2	
*S. gallolyticus*	2	–
Mitis group	78	
*S. gordonii*	5	–
*S. mitis/S. oralis*	12	–
*S. mitis*	12	–
*S. oralis*	24	–
*S. parasanguinis*	18	–
*S. sanguinis*	7	–
Salivarius group	14	
*S. salivarius*	14	–
Reference strains (DSMZ)		
Anginosus group	4	
*S. anginosus*	1	+
*S. constellatus* subsp. *constellatus*	1	+
*S. constellatus* subsp. *pharyngis*	1	+
*S. intermedius*	1	–
Mutans group	1	
*S. mutans*	1	–

*moac, marker of *Streptococcus anginosus* and *S. constellatus;* DSMZ, Deutsche Sammlung für Mikroorganismen und Zellkulturen.

### *Moac* as a Marker for *S. anginosus* within GCGS

The data that were obtained with the Leipzig collection of oral streptococci suggested that *moac* could also be exploited as a marker for β-hemolytic strains of the anginosus group. To further test the quality of *moac* as a marker, the Vellore GCGS collection was subjected to *moac*-specific PCR (*moac*-PCR). The results are summarized in Table 2. All 254 strains that were specified as SDSE on the basis of their 16S rRNA gene sequence were negative in *moac*-PCR. The 59 remaining strains, which could be assigned to the species *S. anginosus* by 16S rRNA gene sequencing, were positive for *moac*. Moreover, strains of the species *S. canis* (*2*), *S. equi* subsp. *zooepidemicus* (*3*), *S. equi* subsp. *equi* (*2*), and *S. dysgalactiae* subsp. *dysgalactiae* (*2*) were negative in the *moac*-PCR, proving that it is a reliable method for identifying anginosus group strains in collections of GCGS. The examination showed that *S. anginosus* isolates constitute 19% of the collection of β-hemolytic GCGS isolates from clinical suppurative infections in Vellore; thus, these pathogens play a considerable epidemiologic role in acute streptococcal infections that occur in this region.

## Discussion

Lancefield groups C and G comprise a variety of distinct species; of these species, *S. dysgalactiae* subsp. *equisimilis, S. equi* subsp. *zooepidemicus, S. canis,* and streptococci of the anginosus group cause severe infections in humans (*2**–**5**, **17**–**27*). Examination of clinical isolates from patients with purulent infections of the upper respiratory tract, the urinary tract, and invasive infections showed that SDSE is dominant in GCGS infections in Vellore, accounting for 81% of the cases. SDSE was responsible for all 7 cases of invasive infections included in this study. *S. anginosus* that possess group G antigens accounted for the remaining 19% of the suppurative infections, which indicates a considerable epidemiologic role for this species in the Vellore region. In contrast, β-hemolytic group C streptococci of the pharyngitis-associated species *S. constellatus* subsp. *pharyngis* (*37*) were not detected. Infections with the typically zoonotic β-hemolytic GCGS *S. equi* subsp. *zooepidemicus* (group C) and *S. canis* (group G) were also not diagnosed, which suggests a comparatively low incidence of such infections in Vellore. Groups C and G antigens are rare in *S. constellatus* subsp. *constellatus* and in *S. intermedius* (*28*), if they are present at all in these species. Their absence in the Vellore GCGS collection, therefore, cannot be considered as indicative that these species have less clinical relevance.

The high phenotypic and antigenic diversity within the anginosus group, and the circumstance that non–β-hemolytic strains remain inconspicuous in samples that contain commensal flora, make anginosus group infections difficult to diagnose. Therefore, contribution of the anginosus group as a whole to the epidemiology of streptococcal infections is poorly understood and needs further investigation, not only in Vellore but also in other regions with suspected high incidence. The required surveys will become feasible with the identification of potent markers for detection of anginosus strains such as the *moac* marker. The isolates from Vellore and Leipzig together represent a highly diverse collection of anginosus strains that differ not only in their geographic origin, but greatly in Lancefield type. The collection comprises strains with groups A, C, G, and F, and without Lancefield antigen. Furthermore, the collection includes isolates with all types of hemolysis. The diagnostic power of *moac* is demonstrated by the herein described PCR test that identified all of the phenotypically diverse *S. anginosus* and *S. constellatus* strains, discriminating them from other streptococci with 100% accuracy. To the best of our knowledge, this PCR method is the only one described that distinguishes *S. constellatus* subsp. *pharyngis* and *S. intermedius*. In addition to the reliability, the use of the moac as a marker may have a further valuable advantage; the *moac* gene is predicted to code for a transmembrane protein, a property that could be exploited for the development of an antibody based test for species determination.

Further valuable diagnostic information may lie in the *emm* gene. Examination of the Vellore collection allowed assessment of *emm* typing for use in species determination. Most of the 254 SDSE strains included in this study were positive for the *emm* gene by PCR. However, 2 strains of this species were negative for it, indicating that a negative *emm*-PCR is not an accurate exclusion criterion in typing of SDSE. In contrast to SDSE strains, all anginosus group strains, from both the Vellore and the Leipzig collection, were negative in *emm*-PCR. This examination of phenotypically diverse strains strongly suggests that a negative result from *emm*-PCR is a common, if not a general, property of the anginosus group. Oligonucleotide hybridization experiments did not show any indications for the presence of *emm*-homologs in such isolates (data not shown). Moreover, and contrary to observations with *S. equi* subsp. *zooepidemicus* and *S. equi* subsp. *equi*, binding experiments with plasma proteins suggest that anginosus strains lack surface proteins that exert the typical functions of M proteins (data not shown).

Despite the absence of this key virulence factor, the anginosus group includes opportunistic pathogens that can cause bacteremia and a variety of severe purulent infections in the oral cavity, the urogenital tract, and internal organs. This circumstance raises the question, what are the factors that allow streptococci of the anginosus group to colonize at the site of infection and to survive under the hostile conditions of the suppurative focus of infection and the bloodstream? Little is known about the pathogenesis of anginosus group infections, but they seem to be governed by unique, unknown mechanisms. Unique mechanisms of pathogenesis and the decreased susceptibility of anginosus group strains to certain antimicrobial drugs (*15*) create a need for well-tailored treatments and thus for accurate diagnosis. In this context, indications that treatment with metronidazole facilitates infections with streptococci of the anginosus group deserve particular attention (*26**,**38**–**40*). Accumulating indications of the considerable clinical relevance of the anginosus group and the propensity of these bacteria to develop antimicrobial drug resistance suggest that they may be a group of emerging pathogens that should be monitored.
